# Local parasite lineage sharing in temperate grassland birds provides clues about potential origins of Galapagos avian *Plasmodium*

**DOI:** 10.1002/ece3.1894

**Published:** 2016-01-11

**Authors:** Iris I. Levin, Rachel E. Colborn, Daniel Kim, Noah G. Perlut, Rosalind B. Renfrew, Patricia G. Parker

**Affiliations:** ^1^ Department of BiologyUniversity of Missouri – St. LouisOne University Blvd.St. LouisMissouri63121; ^2^ Whitney R. Harris World Ecology CenterUniversity of Missouri – St. LouisOne University Blvd.St. LouisMissouri63121; ^3^ Saint Louis ZooWildCare InstituteOne Government Dr.St. LouisMissouri63110; ^4^ Platte River Whooping Crane Maintenance Trust6611 W. Whooping Crane Dr.Wood RiverNebraska68883; ^5^ Department of Environmental StudiesUniversity of New England11 Hills Beach RoadBiddefordMaine04005; ^6^ Vermont Center for EcostudiesPO Box 420NorwichVermont05055; ^7^ Department of Ecology and Evolutionary BiologyUniversity of Colorado – BoulderRamaley N122Box 334BoulderColorado80309

**Keywords:** *Dolichonyx oryzivorus*, Galapagos Islands, haemosporidian parasites, host breadth, migration, *Molothrus ater*, *Plasmodium*

## Abstract

Oceanic archipelagos are vulnerable to natural introduction of parasites via migratory birds. Our aim was to characterize the geographic origins of two *Plasmodium* parasite lineages detected in the Galapagos Islands and in North American breeding bobolinks (*Dolichonyx oryzivorus*) that regularly stop in Galapagos during migration to their South American overwintering sites. We used samples from a grassland breeding bird assemblage in Nebraska, United States, and parasite DNA sequences from the Galapagos Islands, Ecuador, to compare to global data in a DNA sequence registry. Homologous DNA sequences from parasites detected in bobolinks and more sedentary birds (e.g., brown‐headed cowbirds *Molothrus ater*, and other co‐occurring bird species resident on the North American breeding grounds) were compared to those recovered in previous studies from global sites. One parasite lineage that matched between Galapagos birds and the migratory bobolink, *Plasmodium* lineage B, was the most common lineage detected in the global MalAvi database, matching 49 sequences from unique host/site combinations, 41 of which were of South American origin. We did not detect lineage B in brown‐headed cowbirds. The other Galapagos‐bobolink match, *Plasmodium* lineage C, was identical to two other sequences from birds sampled in California. We detected a close variant of lineage C in brown‐headed cowbirds. Taken together, this pattern suggests that bobolinks became infected with lineage B on the South American end of their migratory range, and with lineage C on the North American breeding grounds. Overall, we detected more parasite lineages in bobolinks than in cowbirds. Galapagos *Plasmodium* had similar host breadth compared to the non‐Galapagos haemosporidian lineages detected in bobolinks, brown‐headed cowbirds, and other grassland species. This study highlights the utility of global haemosporidian data in the context of migratory bird–parasite connectivity. It is possible that migratory bobolinks bring parasites to the Galapagos and that these parasites originate from different biogeographic regions representing both their breeding and overwintering sites.

## Introduction

Haemosporidian parasites are increasingly being included in studies of avian ecology and evolution (e.g., Isaksson et al. 2013; Kulma et al. 2013; Larcombe et al. 2013). Molecular screening and lineage identification techniques have contributed to a rapid expansion of data, much of which is publically available. These resources allow for large‐scale comparisons of haemosporidian parasites among hosts and geographic locations (e.g., Marzal et al. 2011; Ewen et al. 2012). Haemosporidian parasites can be useful tools for understanding host ecology, including avian host migratory behavior (Dodge et al. 2013; Svensson et al. 2007; Waldenström et al. 2002; but see Pagenkopp et al. 2008; Fallon et al. 2006). Jenkins et al. (2012) found higher parasite diversity in migratory species, probably because migratory species move between geographically disparate regions and are likely to encounter different vectors and parasite lineages compared to resident species or shorter‐range migrants.

Avian haemosporidian parasites, which require both a vertebrate and arthropod host, include species belonging to the genera *Plasmodium* and *Haemoproteus*. Parasites in both genera have been shown to cause harm to their hosts (Marzal et al. 2005; Lachish et al. 2011), especially in cases where a host encounters a novel parasite (e.g., Atkinson and Samuel 2010; Bueno et al. 2010). Oceanic archipelagos are often considered isolated and potentially more protected from natural colonization by novel pathogens and parasites. However, in addition to one *Plasmodium* lineage repeatedly detected in Galapagos penguins (Levin et al. 2009), we have described three other *Plasmodium* lineages from Galapagos passerine bird samples (Levin et al. 2013). We found that two of the four *Plasmodium* lineages detected in Galapagos birds matched those detected in North American breeding bobolinks (*Dolichonyx oryzivorus*), the only passerine bird species that regularly migrates through Galapagos (Levin et al. 2013). Migratory birds have previously been implicated in the spread of haemosporidian parasites (Valkiūnas 1993; Ramey et al. 2012). In Galapagos, the lineages matching bobolink parasite DNA sequences were each detected at one or two proximate sites at one sampling time point. It is possible that these lineages are not well adapted to Galapagos hosts or vectors, as they have not been detected since their original documentation in Galapagos, despite further sampling. Two other nonmutually exclusive explanations are (1) that the frequency at which these parasite lineages are brought to the islands is too low for transmission to establish and (2) that the spatial–temporal distribution of mosquito and avian host in the islands does not offer opportunities for transmission in a way that is sufficient to sustain Plasmodium transmission.

In this study, we sought to further investigate the potential origins of Galapagos *Plasmodium* lineages, focusing on the two lineages (B and C, Levin et al. 2013) that matched DNA sequences of *Plasmodium* parasites amplified from bobolink samples collected at sampling sites (NE, OR, VT) in the bobolink breeding range in the United States. Bobolinks are long‐distance migrants (20,000 km round trip) that breed across northern North America and overwinter in south‐central South America (for detailed maps see Renfrew et al. 2013). Bobolinks from the same three sampling sites in the United States were tagged with geolocators to characterize the timing and movements associated with their southbound migration (Renfrew et al. 2013). Fifteen geolocators were recovered and, although none of these animals stopped in Galapagos, they showed remarkable spatial and temporal convergence on two sequential sites prior to overwintering in Argentina and Paraguay (Renfrew et al. 2013). While stopping/overwintering at these sites, it is likely that bobolinks acquire local haemosporidian parasites.

We utilized opportunistically collected samples of other local birds at the Nebraska site, one of the three sites used in both the Galapagos parasite lineage matching and the geolocator migratory research. Aside from bobolinks, the local avian assemblage at this grassland site included mostly grasshopper sparrows (*Ammodramus savannarum*), brown‐headed cowbirds (*Molothrus ater*), dickcissels (*Spiza americana*), western meadowlarks (*Sturnella neglecta*), and red‐winged blackbirds (*Agelaius phoeniceus*) (Kim et al. 2008). Brown‐headed cowbirds are a particularly informative species to use in comparison with bobolinks. Both belong to the family Icteridae, so we might expect fewer incompatibilities with haemosporidian parasites due to their recently shared evolutionary histories. Furthermore, unlike bobolinks, brown‐headed cowbirds are short‐distance migrants in the northern part of their range and residents in the southern part (Lowther 1993). Their distribution does not extend into South America, and therefore, they are unlikely to directly encounter parasites whose transmission is restricted to that region.

We compared the haemosporidian parasites detected in brown‐headed cowbirds and other members of the North American grassland breeding bird community to those previously detected in bobolinks. Our haemosporidian lineages were then compared to published global parasite lineages with known geographic sampling locations to begin assessing where the lineages found in bobolinks, especially the two that were detected in Galapagos, were likely transmitted. If a parasite lineage is detected in North American breeding bobolinks, but not brown‐headed cowbirds, and if it matches lineages previously described from South America, we interpret this as evidence for transmission of that lineage on the bobolink overwintering grounds in South America. If a lineage is shared between bobolinks and brown‐headed cowbirds (and perhaps other local species) and matches predominantly North American parasite lineages, it is more likely that the lineage was transmitted on the North American breeding grounds. We predicted higher parasite species richness in bobolinks, which have a greater opportunity of encountering different haemosporidian assemblages than the more sedentary cowbirds. We also predicted greater host breadth for parasites detected in Galapagos, based on other research demonstrating that generalist parasites are more likely to establish in new biogeographic regions (Ewen et al. 2012).

## Methods

### Sampling methodology

Bobolink sampling in Nebraska, Vermont, and Oregon is described in Levin et al. (2013). Brown‐headed cowbirds (*n* = 70), Henslow's sparrows (*Ammodramus henslowii*) (*n* = 15), dickcissels (*S. americana*) (*n* = 6), and one American robin (*Turdus migratorius*) were sampled opportunistically at the Nebraska site between May and July 2002–2011.

### Molecular screening for Haemosporidian DNA

Molecular screening for and sequencing of mitochondrial cytochrome *b* (cyt *b*) for haemosporidian parasites used primers from Waldenström et al. (2004) with a protocol that follows Levin et al. (2013). Parasite DNA sequences were manually checked for evidence of infection with multiple species and any sequence that had evidence of double peaks in the chromatogram was removed from the analyses. We defined parasite (cyt *b*) lineages as any unique parasite DNA sequence at this 490‐bp region of cyt *b*. DNA lineages were matched to sequences in the MalAvi database v.2.1.1 (Bensch et al. 2009) using the integrated BLAST tool. If a 100% lineage match in MalAvi was found, we extracted all host taxonomic and sampling location information. The sequences were compared to those found in bobolinks (reported in Levin et al. 2013), brown‐headed cowbirds and the few other samples of parasites from other grassland bird species (reported here).

### Phylogenetic analysis

We combined our data with sequences of described morphospecies of avian haemosporidian parasites obtained from GenBank. Clustal W (Larkin et al. 2007), implemented in BioEdit v. 7.1.7 (Hall 1999) was used to align sequences. The best fit model of DNA evolution was determined with jModelTest2 (Guindon and Gascuel 2003; Darriba et al. 2012). A GTR + I + Γ model was used to reconstruct a maximum‐likelihood phylogeny and bootstrap analysis (1000 pseudoreplicates) in MEGA 5 (Tamura et al. 2011).

### Parasite lineage richness and host breadth calculations

EstimateS v.9.1.0 (Colwell 2013) was used to calculate a nonparametric estimate of parasite lineage richness (Chao2). Chao2 is the appropriate estimator for the presence/absence data and smaller sample sizes (Durrant et al. 2008) and can be used to determine the total number of haemosporidian lineages expected from the available data. This is accomplished by estimating species accumulation curves based on the sampling data. Unlike rarefaction analysis, which scales to the smaller sample, this method extrapolates to estimate the total number of lineages infecting that species. We calculated Chao2 from 200 randomizations in EstimateS, where individuals were randomized without replacement, using the classic formula for Chao2. We used EstimateS to obtain a rarefied estimate of lineages present in bobolinks if we had sampled 70 individuals. This allowed us to compare the number of parasite lineages between a previously uneven sample of bobolinks (*n* = 438) and brown‐headed cowbirds (*n* = 70).

We computed a standardized host breadth index, $S_{\mathrm{TD}}^{*}$ (Hellgren et al. 2009), that accounts for the number of host species utilized by the parasite, the taxonomic distance among the host species, and the variance of the taxonomic distance among host species. $S_{\mathrm{TD}}^{*}$ is a modification of the index, S_TD_, proposed by Poulin and Mouillot (2003). We compared the host breadth of parasite lineages found in Galapagos to the host ranges of non‐Galapagos lineages. Statistical analyses were performed in Minitab v. 17 (Minitab Inc., State College, PA, USA).

## Results

### Parasite prevalence and lineage identity

**Figure 1:**
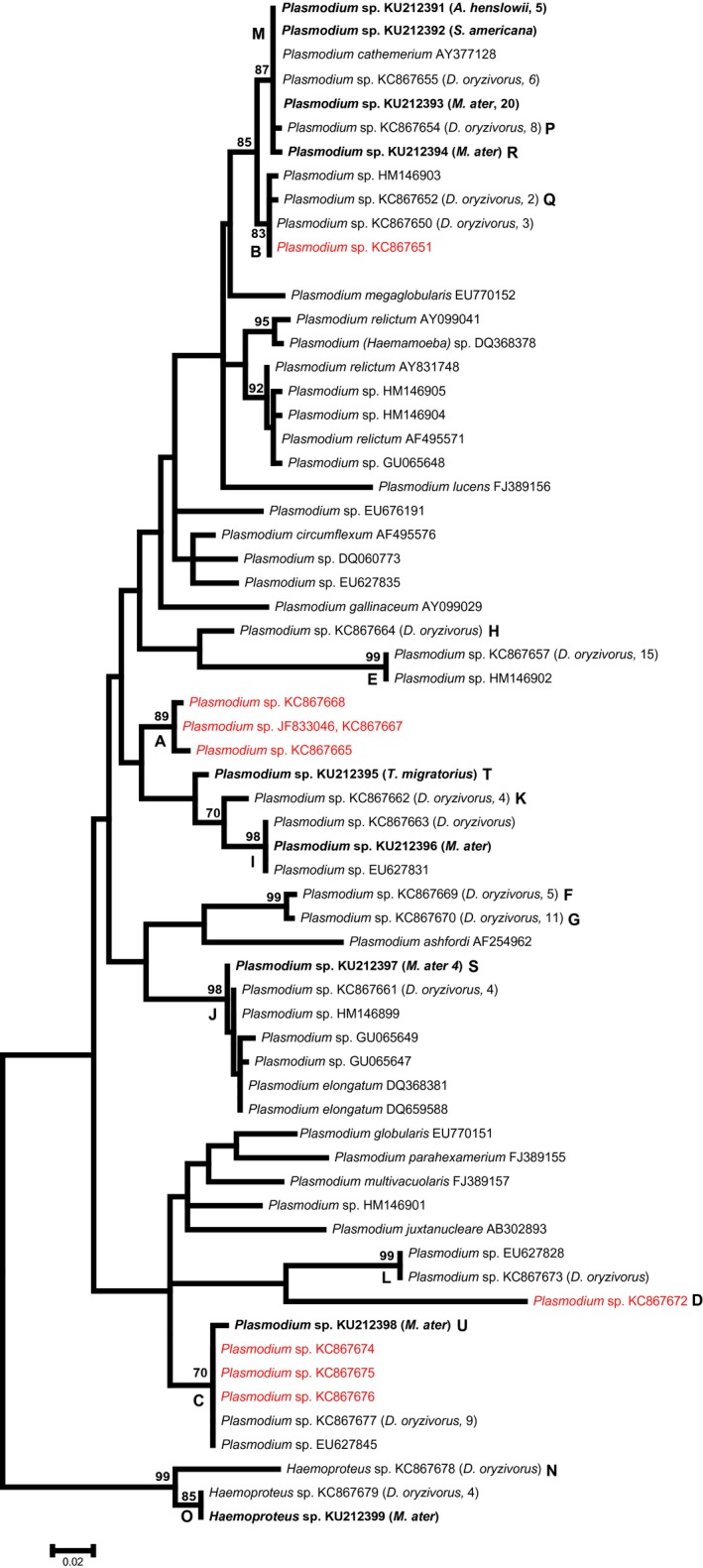
Maximum‐likelihood (ML) phylogenetic hypothesis of haemosporidian parasite DNA sequences constructed from 490 base pairs of the mitochondrial gene; cytochrome *b*. ML bootstrap values appear above the nodes where support values >70 were found. Sequences in red (lineages A–D) are those detected in the Galapagos Islands. Lineages in bold font are those detected in brown‐headed cowbirds (*Molothrus ater*) and other grassland species in this study. Sample sizes of infected individuals follow the host species designation. GenBank accession numbers are listed for all sequences. Lineage letters (A–U) correspond to those in Tables 1, 2.

Prevalence of *Plasmodium* parasites in bobolinks is reported in Levin et al. (2013) but is repeated here for comparison to prevalence in other North American grassland species, with additional information about *Haemoproteus* parasites. We amplified haemosporidian DNA in 78 (17.8%) of the 438 bobolink samples and recovered usable (singly infected) sequence data from 75. Of those 75 sequences, 70 clustered with *Plasmodium* lineages and five were identified as *Haemoproteus* based on sequence similarity. Fifteen lineages were recovered from bobolinks, 13 *Plasmodium,* and two *Haemoproteus* (Tables 1, 2, Fig. 1). Most of the lineages had previously been identified in other studies; only two *Plasmodium* lineages from bobolinks were novel (Table 2). In both cases, these novel lineages were found in more than one individual, so we are confident that they are not DNA sequence errors. We sampled 70 brown‐headed cowbirds and detected 32 infections (45.7% prevalence). Five samples had evidence of multiple infections and were not analyzed further. Of the remaining 27, 26 grouped with *Plasmodium* and one grouped with *Haemoproteus* lineages. In total, we recovered five lineages from brown‐headed cowbirds, four *Plasmodium* lineages, and one *Haemoproteus* lineage (Tables 1, 2, Fig. 1). All of the brown‐headed cowbird lineages matched those from previous studies except for three *Plasmodium* lineages, R, S, and U, found in fewer than four individuals (Table 2). Fifteen dickcissel samples yielded five sequences (33.3% prevalence), which all matched the DNA sequence associated with the *Plasmodium cathemerium* morphospecies (Table 1, Fig. 1). *Plasmodium cathemerium* was also detected in the only parasite DNA sequence from a sample of six Henslow's sparrows (16.7% prevalence). Finally, the one American robin sample was infected with a novel DNA lineage not yet reported in the MalAvi database (lineage T, Table 2). Bobolinks and brown‐headed cowbirds shared only three of the lineages detected (I, M, O, Table 1, Fig. 1).

**Table 1:** Thirteen haemosporidian lineages detected in grassland bird samples (BOBO = *Dolichonyx oryzivorus*, BHCO = *Molothrus ater*, DICK = *Spiza americana*, and HESP = *Ammodramus henslowii*) that match parasite DNA sequences from the previous studies. The MalAvi database (http://mbio-serv2.mbioekol.lu.se/Malavi/) was used to identify 100% sequence matches, to extract the host breadth information, and to identify the geographic region where the lineage had previously been detected. “Percent South American” is a calculation of the proportion of the 100% DNA sequence matches that were detected in that geographic region

Lineage	MalAvi ID	Parasite identity	Host (this study)	No. 100% matches	Host breadth ($S_{\mathrm{TD}}^{*}$)	Geographic region of sequence matches	% S. Am.	References
A (Gal)	SPMEN03	*Plasmodium* sp.	None^a^	1	5.65	S. America	100	6
B (Gal)	PADOM09	*Plasmodium* sp.	BOBO	49	23.83	S. America, N. America, unknown	83.7	1, 9, 10, 18, 19, 21, 23–26
C (Gal)	LAIRI01	*Plasmodium* sp.	BOBO	2	6.49	N. America	0	14, 19, 28
E	PADOM17	*Plasmodium* sp.	BOBO	2	5.00	S. America	100	18, 19, 23
F	VOLJAC02	*Plasmodium* sp.	BOBO	12	13.25	S. America	100	18, 19
G	DENPET03	*Plasmodium* sp.	BOBO	28	26.04	S. America, N. America	85.7	7, 9, 18, 19, 23, 26, 27, 30
H	COLL4	*Plasmodium* sp.	BOBO	8	9.89	S. America, Europe	75	9, 17, 19
I	PHPAT01	*Plasmodium* sp.	BOBO, BHCO	8	10.18	S. America, N. America	62.5	14, 18, 19, 24, 26
J	PADOM11	*Plasmodium* sp.	BOBO	29	25.17	S. America, N. America, Asia, unknown	51.7	3, 9, 14, 16, 18, 19, 21–23, 25
K	WW3/WW7	*Plasmodium* sp.	BOBO	16	15.90	Africa, Europe, N. America	0	2, 4, 5, 10–13, 15, 19, 23, 26, 29, 31
L	RWB01	*Plasmodium* sp.	BOBO	5	7.25	N. America	0	14, 19, 21, 26, 27, 30
M	SEIAUR01	*Plasmodium cathemerium*	BOBO, BHCO, DICK, HESP	27	23.17	N. America, Asia, unknown	0	1, 11, 14, 16, 19–21, 23, 32
N	SIAMEX01	*Haemoproteus* sp.	BOBO	10	12.98	N. America	0	8, 11, 19, 27
O	ICTLEU01	*Haemoproteus* sp.	BOBO, BHCO	1	4.00	Central America	0	19, 25

1, Beadell et al. (2006); 2, Beadell et al. (2009); 3, Beadell and Fleischer (2005); 4, Bensch and Åkesson (2003); 5, Bensch et al. (2012); 6, Carlson et al. (2011); 7, Chagas et al. (2013); 8, Donovan et al. (2008); 9, Durrant et al. (2006); 10, Durrant et al. (2007); 11, Ferrell et al. (2007); 12, Hellgren (2005); 13, Hellgren et al. (2007); 14, Ishak et al. (2008); 15, Ishtiaq et al. (2012); 16, Kimura et al. (2006); 17, Kulma et al. (2013); 18, Lacorte et al. (2013); 19, Levin et al. (2013); 20, Loiseau et al. (2012); 21, Martinsen et al. (2007); 22, Martinsen et al. (2008); 23, Marzal et al. (2011); 24, Merino et al. (2008); 25, Outlaw and Ricklefs (2009); 26, Pagenkopp et al. (2008); 27, Ricklefs and Fallon (2002); 28, Schrenzel et al. (2003); 29, Synek et al. (2013); 30, Szymanski and Lovette (2005); 31, Waldenström et al. (2002); 32, Wiersch et al. (2005).

a Previously reported infecting Galapagos penguins (Levin et al. 2009).

**Table 2:** Novel parasite DNA lineages detected in *Dolichonyx oryzivorus*,*Molothrus ater*, and *Turdus migratorius*

Lineage	Parasite identity	Host (this study)	No. infected individuals
P	*Plasmodium* sp.	*Dolichonyx oryzivorus*	8
Q	*Plasmodium* sp.	*Dolichonyx oryzivorus*	2
R	*Plasmodium* sp.	*Molothrus ater*	1
S	*Plasmodium* sp.	*Molothrus ater*	4
T	*Plasmodium* sp.	*Turdus migratorius*	1
U	*Plasmodium* sp.	*Molothrus ater*	1

We found substantial variation in the frequencies of different DNA lineages across the dataset and among host species. The most common lineage, M, was the only one we could tentatively assign to a morphospecies (*P. cathemerium*). We sequenced *P. cathemerium* 32 times, including 20 of the 27 single infections in brown‐headed cowbirds and from all infected dickcissels and Henslow's sparrows. The next most frequently detected lineages were lineages E (*n* = 15), G (*n* = 11), and C (*n* = 9). Lineage C is one of the two lineages found in Galapagos that matched parasite DNA sequences from bobolinks (Levin et al. 2013). Over half the lineages were found in five or fewer samples.

### Phylogenetic analysis

The phylogenetic tree (Fig. 1) shows little support for most of the relationships within *Plasmodium*. Some groups do have bootstrap support >70, including sequences matching or closely related to *P. cathemerium*. This particular clade, which includes lineage B, M, P, Q, and R, is further split into two well‐supported clades. One clade contains parasites found in bobolinks, Q, and in bobolinks and Galapagos passerines, B. Sister to that clade is the one including *P. cathemerium* and variants that differ by a few base pairs. The relationships between the clades containing *P. relictum*, a parasite lineage of conservation concern due to its contribution to avian extinctions in Hawaii, are less clear. However, no Galapagos lineages fall within the well‐supported groups containing *P. relictum*.

Although we cannot resolve many of the relationships within the phylogeny, we were more interested in understanding the geographic scope of each lineage rather than understanding the complicated systematics of this group. Clades do not necessarily correspond to biogeographic regions; most clades contain lineages found in both North and South America (see Table 1).

#### Putative geographic origins of Galapagos *Plasmodium* lineages

We had previously described two perfect parasite DNA matches between *Plasmodium* sequences from Galapagos passerine birds and bobolinks (Levin et al. 2013). In order to begin inferring the origins of these Galapagos sequences, we analyzed the geographic locations of every sequence that matched a lineage detected in our study. Table 1 shows the geographic regions where each lineage has been reported and displays these data as a percentage of the sequences that were detected from samples collected in South America. Lineage B, detected in Galapagos yellow warblers (*Setophaga petechia*) and bobolinks sampled in Vermont, matched 49 other sequences in the MalAvi database recovered from 22 species. Forty‐one (84%) of those sequences were detected in birds sampled in South America. In our study, this lineage was detected in bobolinks but not brown‐headed cowbirds, consistent with transmission of this parasite in bobolink wintering sites in South America.

Lineage C, the other perfect match between parasites of bobolinks and Galapagos birds (Levin et al. 2013), matched two other sequences in MalAvi, detected in two different species from two North American sampling sites (Table 1). Although this lineage was not detected in our brown‐headed cowbird samples, a very close variant lineage (lineage U, 1 bp different) was recovered in this species (KU212398, Table 2), providing some evidence for transmission at North American breeding sites of bobolinks and cowbirds.

There was no significant difference in the proportion of sequences that were of South American origin between lineages found in bobolinks and those shared with brown‐headed cowbirds (Kruskal–Wallis test, *χ* ^2^ = 0.01, df = 1, *P* = 0.91). However, Table 1 shows that lineages found in bobolinks but not brown‐headed cowbirds are, with one exception, either entirely North American lineages or lineages that are predominantly detected in South America. This suggests that bobolinks are infected by parasite lineages common to both North and South America and are likely exposed to distinct lineages while on the North American breeding grounds and their South American overwintering grounds.

### Parasite lineage richness and host breadth

We did detect many more parasite lineages in bobolinks (15) than in brown‐headed cowbirds (6); however, the sample sizes are heavily skewed toward bobolinks. In order to ensure we were not biasing our interpretations of lineage richness in favor of the better‐sampled host species, we used two alternative measures of estimating the number of parasites lineages in each host. We performed a rarefaction analysis on the bobolink data using the sample size for cowbirds (*n* = 70). Results from the rarefaction analysis showed twice as many lineages in bobolinks (13.3 lineages) compared to the observed 6 in the same sample of brown‐headed cowbirds (Table 3). However, the standard deviation of this rarefaction calculation is large and there is a small amount of overlap in the confidence intervals for the estimates. An alternative way to compare lineages in these hosts is to calculate parasite lineage richness (Chao2), shown in Table 3. Note that the 95% confidence intervals of the Chao2 estimate overlap substantially. This analysis suggests that we may have missed a few lineages in bobolinks (15 lineages observed, 95% CI lower bound = 18.27), but that our six observed lineages in brown‐headed cowbirds is close to the 95% CI lower bound of 6.95) (Table 3).

**Table 3:** Estimated expected lineage richness (Chao2), observed number of lineages, and, in the case of the *Dolichonyx oryzivorus* sample, a calculation of the rarefied number of parasite lineages using the *Molothrus ater* sample size

Host species	*Dolichonyx oryzivorus*	*Molothrus ater*
Sample size	438	70
Observed parasite lineages (rarefied, *n* = 70)	15 (13.3 ± 8.1)	6
Estimated lineage richness ± SD (Chao2)	29.96 ± 13.59	11.91 ± 6.95
95% CI lower bound	18.27	6.95
95% CI upper bound	83.46	42.7

We were also interested in the host breadth of each parasite lineage, particularly in comparing the host breadth of the parasites found in Galapagos to those found elsewhere. We compared the host breadth of Galapagos lineages that occurred in more than one sample (*n* = 3) to the host breadth of non‐Galapagos lineages detected in our study (*n* = 11). We did not find support for our prediction that Galapagos lineages had larger host breadth than non‐Galapagos lineages (*t*‐test, *T* = 1.24, df = 2 *P* = 0.34). Additionally, there was no difference in host breadth for lineages found in bobolinks (*n* = 12) vs. lineages found in both bobolinks and brown‐headed cowbirds (*n* = 3) (*t*‐test, *T* = 0.08, df = 2, *P* = 0.942). Lineage G had the greatest host breadth ($S_{\mathrm{TD}}^{*}$ = 26.04), while lineage O had the narrowest host breadth ($S_{\mathrm{TD}}^{*}$ = 4.00, all Icteridae hosts). The three Galapagos lineages A, B, and C had $S_{\mathrm{TD}}^{*}$ values of 5.65, 23.83, and 6.49, respectively.

## Discussion

This study provides insights into the origins of two Galapagos *Plasmodium* lineages by utilizing host sampling location information associated with DNA sequence matches. Additionally, we gain useful information from a comparison of parasite sequences in bobolinks, a migratory host vs. a more sedentary but related species, the brown‐headed cowbird, which co‐occurs with bobolinks on the North American breeding grounds. Although our comparison of haemosporidian parasites in migratory vs. more sedentary hosts is restricted to the Nebraska sampling site, we did not previously find significant differences in parasites lineage frequency between sites (Levin et al. 2013).

Lineage B, detected in Galapagos yellow warblers and breeding bobolinks sampled in Vermont (with a 1‐bp different variant sampled in Nebraska), is a fairly common *Plasmodium* lineage (previously reported from 49 other host/site combinations) found predominantly in South America, suggesting that this lineage may have been acquired by bobolinks while overwintering in South America. The only other North American samples in which this lineage has been detected are from two warbler species, black‐and‐white warbler (*Mniotilta varia*; Martinsen et al. 2007) and the common yellowthroat (*Geothlypis trichas*; Pagenkopp et al. 2008), which are also long‐distance migrants that overwinter in Central America, the Caribbean and the northern part of South America. We did not have samples from short‐distance migrants or resident birds that co‐occur with bobolinks in Vermont. Lack of detection of lineage B in Vermont residents/short‐distance migrants would strengthen our inference.

The other Galapagos and bobolink *Plasmodium* lineage whose origin we seek to understand, lineage C, is rarely reported in other studies. There are only two matches, one from a captive bird at the San Diego Zoo (Schrenzel et al. 2003) and the other from a barred owl (*Strix varia*), also sampled in California (Ishak et al. 2008). This suggests that if migrating bobolinks did introduce this lineage to the Galapagos Islands, they most likely acquired this parasite in their North American breeding grounds. Confirmation of this would require sampling of juvenile bobolinks and brown‐headed cowbirds. If these individuals, who have yet to migrate to other regions, are infected with the same *Plasmodium* lineage, we can confirm transmission in that area. One must keep in mind that this approach is only as good as the available data and potentially subject to the sampling biases inherent in the data. For example, the MalAvi database has far more samples from S. America compared to N. America (Clark et al. 2014), which could affect our interpretations in our study. More sampling (particularly in N. America), sequencing of the same region of parasite cyt *b*, and continued support for databases like MalAvi will allow for further refinement of these hypotheses in the future. Additionally, blood smears for parasite morphological data would be a valuable addition.

We found three haemosporidian lineages shared between bobolink and cowbird hosts (2 *Plasmodium*, 1 *Haemoproteus*). Bobolinks were found infected with exclusively North American lineages or, with one exception, lineages that were predominantly found at South American sampling sites. In another multispecies study, Waldenström et al. (2002) found that nearly half of the lineages detected were present in multiple hosts, including resident and migratory species. However, they did also find clades that supported sole transmission in one region or the other (Waldenström et al. 2002). Our results suggest that one lineage, H, might be transmitted both on the North American breeding grounds and in South America. Lineage H infects bobolinks and brown‐headed cowbirds in Nebraska, and 37% of the DNA sequence matches in MalAvi are parasites of North American birds. The remaining 63% are of South American origin. This lineage was detected in other North American birds that either have a distribution that extends into South America (Great Horned Owl, *Bubo virginianus*; Ishak et al. 2008) or in common yellowthroats (Pagenkopp et al. 2008), for which the overwintering range includes the northern part of South America. It is important to note that while widespread lineages might suggest contemporary gene flow among parasite populations, they might also reflect the lack of sequence variation in *Plasmodium* cyt *b* (Hellgren et al. 2013; Clark et al. 2014).

Although there is a slight overlap in the confidence intervals of the parasite lineage estimates, our rarefaction analysis revealed that more haemosporidian lineages were likely in bobolinks than in brown‐headed cowbirds. This is consistent with other research that finds greater parasite diversity in migratory birds compared to nonmigratory birds (Jenkins et al. 2012) and in birds with migratory divides (two geographically distinct overwintering sites) compared to those without (Møller et al. 2011). We cannot ignore the arthropod hosts required for successful transmission when considering shifts in haemosporidian parasite distributions due to migratory birds. Whether or not a parasite can establish in a new biogeographic region will depend on the competence and feeding preferences of the local arthropod vectors. Furthermore, migratory species might have a relatively extended period of exposure to vectors as they are potentially exposed to vectors throughout the year. Recently, host compatibility, rather than the rate at which vectors encounter hosts, has been shown to better explain the distribution of *Plasmodium* parasites among bird host species (Medeiros et al. 2013).

Our estimate of parasite lineage richness indicates that we may be missing lineages occurring in bobolinks. The observed number of lineages, 15, is lower than the 95% confidence interval for the expected parasite lineage richness (Chao2) of 18.27 (Table 3). This is most likely due to the substantial proportion of rare lineages detected in bobolink samples. Although our sample of brown‐headed cowbirds is much smaller than our sample of bobolinks, our observed parasite lineage number in cowbirds approaches the lower bound of the 95% confidence interval, indicating that we did capture a reasonable number of lineages despite the difference in sampling effort. In both cases, particularly in bobolinks, we are likely still underestimating the number of parasite lineages found in these birds. The high estimate for lineage number in bobolinks could be due to the larger number of rare lineage variants. Besides their wintering migration, an additional explanation could be the unusual nonbreeding movements of bobolinks. Bobolinks utilize three geographically disparate regions during the nonbreeding season (approximately 1 month in Venezuela, 1 month in northern Bolivia and then more than 1 month farther south; Renfrew et al. 2013), and this behavior may result in a higher probability of acquiring local haemosporidian lineages in each location.

Our comparison of host breadth between Galapagos *Plasmodium* lineages and non‐Galapagos haemosporidian lineages detected in bobolinks, brown‐headed cowbirds, and other grassland species revealed similar host breadth in the Galapagos lineages. This result is inconsistent with Ewen et al.'s (2012) finding regarding the establishment of exotic *Plasmodium* lineages in New Zealand. There, the successfully invading lineages were more likely to be global generalists with geographically widespread distributions and had evidence for compatibility with a broad taxonomic range of hosts (Ewen et al. 2012). Furthermore, all of the invading lineages were recorded infecting introduced birds in New Zealand (Ewen et al. 2012). In the case of Galapagos, three of the four lineages were detected in two or three highly localized samples at only one time point (Levin et al. 2013). Additionally, we have evidence of abortive parasite development that suggests that these *Plasmodium* lineages may not be completing their life cycles in endemic Galapagos birds (Levin et al. 2013). Therefore, because we tested such a large sample of Galapagos birds for haemosporidian parasites (>3500), we may have encountered cases where parasite lineages arrived to Galapagos via migratory birds but did not establish, perhaps because the parasite species is not a generalist or because a sufficiently abundant competent vector does not reside in the islands, or at the site of arrival of the parasite. Understanding the parasite community in the Galapagos and the parasites harbored by species that stop in the Galapagos during migration is an important part of conservation efforts. This information is critical because we detect parasites in the 10% of samples from endangered Galapagos penguins (Levin et al. 2009), and we have evidence from antibodies that significantly more than 10% are exposed to *Plasmodium* (Palmer et al. 2013). A stochastic, individual‐based model that includes the effects of *Plasmodium* suggests that the parasite could drastically reduce the probability of penguin population persistence over the next 100 years (Meile et al. 2013).

In conclusion, the Galapagos Islands serve as a fall stopover site for migrating bobolinks. By comparing the haemosporidian parasite lineages detected in bobolinks to resident birds/shorter‐distance migrants, we are able to begin teasing apart potential origins of two Galapagos *Plasmodium* lineages. One lineage is most likely transmitted in South America, where bobolinks overwinter, while the other appears to be a North American lineage transmitted during the breeding season. There are still many more questions about Galapagos avian *Plasmodium* parasites than answers, but this study highlights the utility of global haemosporidian data in the context of migratory bird–parasite connectivity.

## Conflict of Interest

None declared.
